# Hypereosinophilic Syndrome Complicated by Eosinophilic Myocarditis With Dramatic Response to Steroid

**DOI:** 10.1177/2324709618764512

**Published:** 2018-03-19

**Authors:** Mazin Khalid, Vijay Gayam, Sumit Dahal, Ebad Ur Rahman, Kamal Fadllala, Jaspreet Kaler, Mowyad Khalid, Alix Dufresne

**Affiliations:** 1 Interfaith Medical Center, Brooklyn, NY, USA; 2 Detroit Medical Center, Wayne State University, Detroit, MI, USA

**Keywords:** eosinophilia, myocarditis, steroids, hypereosinophilic

## Abstract

*Introduction.* Eosinophilic myocarditis is an infiltrative disease that affects the myocardium leading to various presentations. It can be precipitated by medications, helminthiasis, or hypereosinophilic syndrome. *Case.* We present the case of a young, male patient who presented with palpitations and dyspnea and was found to have heart failure with reduced ejection fracture of 12%. His past medical history was significant for recent lung problem treated with steroids. Based on his history and laboratory findings, he was started on intravenous steroids for treatment of eosinophilic myocarditis. Within 3 days, his ejection fracture improved to 35%. *Conclusion.* Given the nonspecific clinical presentations, mimicking other diseases, high index of suspicion is warranted to diagnose eosinophilic myocarditis. This is crucial as early detection and treatment with steroids can lead to a dramatic response.

## Case Report

A 34-year-old man of Middle Eastern descent presented to the emergency room of the hospital with a 3-day history of generalized myalgia, arthralgia, and fatigue. He described the pain as being dull, located in almost all the joints of the appendicular skeleton, and was associated with gross restriction of movement. His pain worsened with movement. On the day of presentation, he started to experience palpitations and exertional dyspnea. He denied any associated chest pain, orthopnea, pedal edema, nausea, vomiting, sweating, and light headedness or dizziness. He reported travelling to Morocco 6 months prior where he worked in the countryside. He denied any skin rash, tick/insect bites, diarrhea, fevers, chills, cough, flu-like symptoms, or exposure to contaminated water sources. He denied any significant past medical history. The patient denied any smoking, alcohol use, or illicit drug use, and had no known drug allergy. He was, however, admitted to another medical facility 3 months ago where he was treated for eosinophilic pneumonitis. The diagnosis had been established by bronchoscopy and tissue biopsy. He was discharged on a tapering regimen of oral prednisone.

At arrival to our emergency department, his temperature was 98.1°F, blood pressure was 122/90 mm Hg, and pulse rate was 122 beats per minute. He was in acute distress secondary to the palpitation. His estimated jugular venous pressure was about 7 cm H_2_O. Examination of the lungs revealed no wheezing or rales with normal vesicular breath sounds in all zones. Cardiac examination revealed tachycardia with regular heart rate and no murmurs or rubs. No peripheral edema of the lower extremities was noted. The rest of the physical examination was normal.

**Figure 1.**
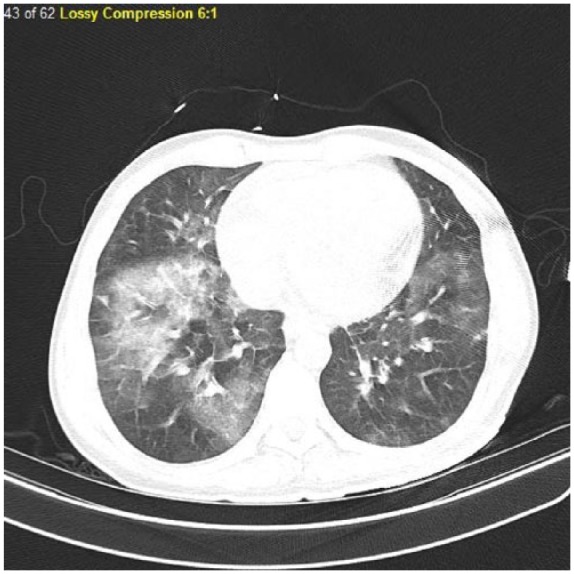
Computed tomography of the chest showing bilateral infiltrates attributed to eosinophilic pneumonia.

**Figure 2.**
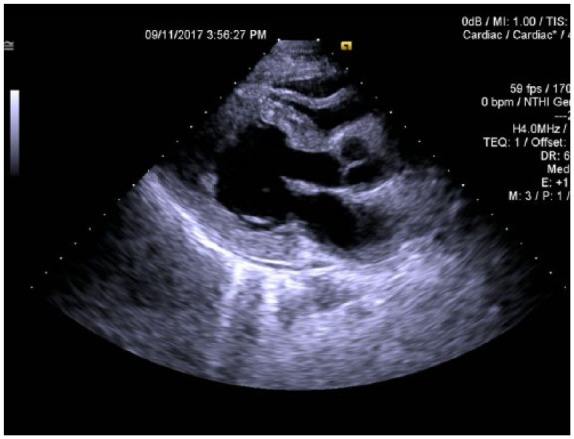
Echocardiography showing dilated ventricle in systole.

Laboratory testing revealed the patient’s white blood cell count of 22.4 × 10^3^/µL, hemoglobin of 12.3 g/dL, hematocrit of 38.3%, and platelet count of 253 × 10^3^/µL. Differential analysis showed 56% of eosinophils with an absolute count of 12 700/µL. Comprehensive metabolic panel revealed no notable abnormalities. His serum troponin I was 22.8 ng/mL (normal value <0.05 ng/mL), and his β-natriuretic peptide was 981 pg/mL (normal value = 0-100 pg/mL). Electrocardiography findings showed sinus tachycardia with no ST-T wave abnormalities. Chest X-ray showed the presence of patchy right lower lobe infiltrate while his chest computed tomography scan showed small anterior pericardial effusion and moderately extensive patchy bilateral ground glass opacities (Figure 1). Emergency echocardiography revealed severe systolic dysfunction and left ventricular dilation with an ejection fraction of 12% (Figure 2).

Apical hypokinesis was noted with questionable intramural thrombus. His right ventricle was normal in size and systolic function with systolic pressure of 36 mm Hg. No valvular abnormality was noted. No pericardial effusion was visible. Given the above findings on the complete blood count, elevated troponins, electrocardiogram, and echocardiogram changes, a presumptive diagnosis of eosinophilic myocarditis (EM) was made, and he was given a single dose of 125 mg of methylprednisolone followed by 60 mg every 8 hours besides aspirin, clopidogrel, enoxaparin, metoprolol, enalapril, and furosemide.

On the second day of his admission, his symptoms started to improve while his peripheral eosinophilia reduced markedly. Steroids were continued on high doses with tapering regimen while monitoring complete blood count for rebound eosinophilia on a daily basis. Troponin was also trended and was noted to decrease slowly. On the third day of hospitalization, the repeat echocardiogram showed an increase in the ejection fraction to 30% to 35%. He underwent cardiac catheterization, which showed patent coronary arteries and ejection fraction of 35%.

He responded well to the treatment and his symptoms were resolved. An extensive workup for the cause of his eosinophilia showed elevated immunoglobulin E levels (4467 IU/mL, reference range = 0-100 IU/mL) and C-reactive protein 167.5 mg/L (upper limit of normal = 4 mg/L). Further workup included negative antinuclear antibodies, negative antineutrophil cytoplasmic antibodies, negative JAK2 mutation analysis, and negative flow cytometry for immunophenotypically abnormal T-cells associated with lymphocytic hypereosinophilic syndrome. His stool studies and parasite serologies for strongyloides was also negative. With all other causes for eosinophilia ruled out, he was diagnosed to have hypereosinophilic syndrome and discharged home on an alternate day dosing of 20 mg of oral prednisone.

## Discussion

Eosinophilic myocarditis was first described in the literature by Wilhelm Loffler in 1936. In his observations, he described endocarditis parietalis fibroplastica and peripheral eosinophilia.^1^ EM is characterized by diffuse or focal inflammation with eosinophilic infiltration, which is often associated with peripheral eosinophilia.^2^ Eosinophilia can be attributed to various disorders and etiologies. Common causes of eosinophilia include infectious, allergic, neoplastic, hematological disorders and other less common conditions.^3,4^ Of these notable causes, myocarditis secondary to medication hypersensitivity have been recognized as the most common cause.^5^

EM is characterized histopathologically by eosinophils and lymphocytes infiltration. The eosinophils degranulate and release toxic granules.^6^ Hypereosinophilic syndrome (HES) has been defined as eosinophilia >1500/mm^3^ for more than 6 months, without any secondary cause and with evidence of tissue damage.^7^ Our patient had not been taking any medications and had no personal or familial history of allergic diseases. His stool and rheumatological workup was negative. In view of his laboratory findings and his previous lung biopsy, we assumed that he had HES complicated by EM.

Classically, EM is divided into 3 chronological stages: eosinophilic infiltration, thrombosis, and fibrosis.^8^ Patients with EM may present with various symptoms ranging from palpitations, fever, chest pain, and shortness of breath to heart failure and cardiogenic shock.^9,10^ The gold standard noninvasive modality for diagnosis of EM is cardiac magnetic resonance imaging; however, it is not widely available and costly. Endomyocardial biopsy remains the best modality to reach a definitive diagnosis.^11^ In our patient, we had all the necessary data to establish a diagnosis of HES. We opted to start the patient’s treatment based on clinical diagnosis without diagnostic testing given the available clinical data.

Steroids are the standard treatment for HES and EM; however, there is no clear consensus on the dosage. Ogbogu et al conducted a retrospective study on 188 subjects, out of which 163 received steroids with a median dose of 40 mg of prednisone. According to this study, 85% of the patients experienced complete or partial response after 1 month of treatment.^12^ The therapy consists of standard heart failure medication in adjunction to corticosteroids.^13,14^ Wong et al demonstrated complete recovery and normalization of cardiac contractility after treatment with high-dose steroids with gradual tapering.^15^ Tyrosine kinase inhibitors, for example, imatinib, have also been shown to have efficacy in patients with myeloproliferative variants with *FIP1L1-PDGFRa* mutations.^16^ Aggarwal et al indicated adjunct azathioprine (2 mg/kg) in patients presenting with EM especially with cardiogenic shock.^17^ Other treatments reported to be effective are hydroxyurea, interferon-α, cyclosporine, and anti-IL-5 antibody (mepolizumab) therapy.^12^ Immunosuppression with cyclophosphamide was reported in treatment of EM that is attributed to vasculitis, namely, Churg-Strauss disease.^18^ In our patient, we elected to start methyl prednisolone 125 mg intravenous bolus followed by 40 mg intravenously every 8 hours. Our patient’s ejection fraction doubled in 3 days, which is a shorter duration compared with previous studies.^19^ Anticoagulation should be considered in acute settings given the risk of endomyocardial thrombus formation,^20^ especially in people with significantly reduced systolic function. However, the necessity and timing of starting anticoagulation remains controversial.

## Conclusion

Given the various clinical presentations, high index of suspicion is warranted when approaching patients with eosinophilia, especially if they have history of organ involvement. This is even more important, as early detection and treatment with steroid can lead to dramatic response improving the patient’s prognosis.
